# Transformation profiles of the isoflavones in germinated soybean based on UPLC–DAD quantification and LC–QTOF–MS/MS confirmation

**DOI:** 10.1016/j.fochx.2024.101413

**Published:** 2024-04-24

**Authors:** Hong Chen, Rezeye Aili, Manyuan Wang, Feng Qiu

**Affiliations:** Beijing Key Lab of TCM Collateral Disease Theory Research, School of Traditional Chinese Medicine, Capital Medical University, Beijing 100069, China

**Keywords:** Germinated soybean, Isoflavones, Transformation, Sprout length, UPLC–DAD, LC–QTOF–MS/MS, Daidzein (PubChem CID: 5281708), Daidzin (PubChem CID: 107971), Genistein (PubChem CID: 5280961), Genistin (PubChem CID: 5281377), Glycitein (PubChem CID: 5317750), Glycitin (PubChem CID: 187808), 6”-*O*-malonyldaidzin (PubChem CID: 9913968), 6”-*O*-malonylgenistin (PubChem CID: 15934091), 6”-*O*-acetylglycitin (PubChem CID: 10228095)

## Abstract

Germinated soybean is one kind of food and a medicine. In the actual process of producing a large amount of naturally germinated soybean, it is difficult to strictly control the germination process conditions. However, sprout length may be more suitable as the terminal judgment indicator for naturally germinated soybean. An UPLC–DAD method was developed and validated to explore the transformation profiles of soybean isoflavones in germinated yellow or black soybean with different sprout lengths. Moreover, an LC − QTOF−MS/MS method was used to avoid false positive results. The contents of daidzein, glycitein, and genistein almost reached their corresponding maximum values when the sprout length ranged from 1.0 cm to 1.5 cm (*P* *<* *0.05*). Therefore, yellow soybean is suggested to be the processing raw material with higher contents of those isoflavones, and the optimal sprout length for germinated soybean may be in the range of 1.0–1.5 cm.

## Highlights

• Six isoflavone glycosides and three isoflavone aglycones had been quantified by a developed UPLC-DAD method.
• An LC − ESI − QTOF−MS/MS method was developed to avoid false positive results.
• Sprout length is more suitable as the terminal judgment indicator for naturally germinated soybean.
• The optimal sprout length for germinated soybean is in the range of 1.0–1.5 cm.

## 1 Introduction

Soybean foods have been dietary staples with high nutritional value, and involve a crucial role in traditional diets around the world such as germinated soybean, Semen Sojae Praeparatum, Natto, fermented soybean milk and soybean sauce. It is beneficial for people to consume soybean, including improved menopause syndrome and reduced the risk of obesity, cardiovascular pathology, specific cancer and immune disorders (Cao, Green-Johnson, Buckley, & Lin, 2019; Chatterjee, Gleddie, & Xiao, 2018**;** Hu, Wong, Wu, & Lai, 2020). Due to the benefits of soybean and its related products, they are often consumed around the world as food, medicine and health care products (Dong et al., 2021). It is well known that fermentation bestows soybean unique flavor that maybe unacceptable to some people. Moreover, the types and contents of those compounds in soybean maybe vary greatly after fermentation, and the safety evaluation of fermented soybean products has been limited (Cao et al., 2019; Inoue et al., 2016**;** Jayachandran & Xu, 2019).

Many studies have demonstrated that germination is a cheap and available way to improve the nutritional quality of legumes as it increases the contents of easily absorbable isoflavone aglycones, amino acids, total dietary fibers, and total soluble sugars, while reducing antinutrient levels such as α-galactosides (Chatterjee et al., 2018; Dong et al., 2021; Hu et al., 2020). Germination is also an effective natural process to enrich polyphenolic, isoflavone aglycones and linked to antioxidative effects (Quinhone & Júnior Ida, 2015). It's well known that soybean isoflavones show estrogen-like effects. People can reduce cardiovascular disease, breast and prostate cancer, menopausal symptoms, and bone loss by consuming soybean isoflavones (Hu et al., 2020; Ma et al., 2020**;** Zaheer & Humayoun Akhtar, 2017). The prevention and treatment of chronic diseases is a long-time process, so long time use may be required. Compared with the efficacy of fermented soybean products, that of germinated soybean is milder, so the long-term use of germinated soybean may be safer and more controllable. Therefore, germinated soybean may be a better choice for patients with chronic diseases.

In general, the chemical components in germinated soybean are similar with those in raw soybean, such as isoflavones, proteins, soyasaponins, amino acids, phytosterols (Dia et al., 2012; Gu et al., 2017**;** Lin & Lai, 2006**;** Ma, Wang, Yang, Zhou, & Gu, 2019**;** Zahir, Fogliano, & Capuano, 2021). But there are also some significant differences, especially the ratios between isoflavone glycosides and isoflavone aglycones. Soybean isoflavones could be divided into several groups, including malonyl isoflavone glycosides (6”-*O*-malonyldaidzin, 6”-*O*-malonylgenistin), acetyl isoflavone glycoside (6”-*O*-acetylglycitin), glucose isoflavone glycosides (daidzin, genistin, glycitin) and isoflavone aglycones (daidzein, genistein, glycitein). The proposed isoflavone biosynthesis pathway in soybean was shown in Fig. S1. After germination, the types (isoflavone glycosides and isoflavone aglycones) and the contents of those compounds have changed to a certain extent, such as the isoflavone glycosides contained in soybeans are partially converted into isoflavone aglycones, which are more conducive to direct absorption and utilization by the body and are more beneficial to human health. Soybean isoflavones are the main active ingredient in soybean and have obvious pharmacological activity, so the change of their types and contents may lead to a great change in pharmacological effects. Therefore, as one kind of medicine, germinated soybean should require a more rigorous and precise process, rather than simply being used as a food using a broad process, such as a fixed time for germinating (Wu et al., 2012). The slight changes of germination environment, such as temperature and humidity, may lead to the sprout length of products is different (Hourston et al., 2020). And the difference leads to different chemical composition, which ultimately leads to a difference in efficacy. Therefore, it is necessary to explore the transformation profiles of those active compounds in germination, to standardize the process of germinated soybean and obtain stable and uniform products. In the actual process of producing a large amount of naturally germinated soybean, it is difficult to strictly control the germination process conditions, such as temperature, humidity, etc. Sprout length may be more suitable as the terminal judgment indicator for naturally germinated soybean. However, it is still unknown whether black soybean or yellow soybean is better as raw materials for sprouting, and what length of sprouting is more suitable for consumption and medicinal purposes.

Therefore, to explore the transformation profiles of soybean isoflavones in germinated yellow and black soybeans and in germinated soybean with different sprout length, a selective, accurate and reliable UPLC–DAD (ultra performance liquid chromatography coupled with diode array detection) method was developed and validated, and a LC − QTOF−MS/MS (liquid chromatography coupled with electrospray ionization and quadrupole time–of–flight tandem mass spectrometry) method was further administrated to avoid false positive results.

## 2 Materials and methods

### 2.1 Chemicals and materials

Yellow soybean and black soybean were both purchased from local supermarket (Beijing, China). All these soybean samples were authenticated to be *Glycine* max (L.) Merr. by Associate Professor Rong Luo (Capital Medical University, Beijing, China) according to the Pharmacopoeia of the People's Republic of China (Chinese Pharmacopoeia Commission, 2020).

Reference standards of daidzein (batch no. 11502–200,402), daidzin (93.3%, batch no. 111738–201,603), genistein (99.5%, batch no. 111704–201,703) and genistin (99.9%, batch no. 111709–201,702) were purchased from National Institutes for food and drug control (Beijing, China). Reference standards of glycitein (>98.0%, batch no. 11502–200,402) was purchased from Beijing Bailingwei technology co., ltd. (Beijing, China). Reference standards of glycitin (>98.0%, batch no. G25A1484), 6”-*O*-malonyldaidzin (>95.0%, batch no. BE17B124) and 6”-*O*-malonylgenistin (>98.0%, batch no. BD24B001) were purchased from Chengdu push bio-technology co., ltd. (Beijing, China). Reference standard of 6”-*O*-acetylglycitin (>98.0%, batch no. P20J9F66220) was purchased from Shanghai Yuanye bio-technology co., ltd. (Shanghai, China). The structures of these nine soybean isoflavones were shown in Fig. S2

Methanol (LC − MS grade), acetonitrile (LC − MS grade grade) and dimethyl sulfoxide (analytical grade) were purchased from Fisher (Marshalltown, IA, USA). Formic acid and other solvents were analytical grade from Sinopharm Group Chemical Reagent Co., Ltd. (Beijing, China).

### 2.2 Instruments

UPLC analysis was carried out on an Agilent 1260 series UPLC system (Agilent, USA), and the column used was an ACQUITY UPLC® HSS 2.1 mm × 100 mm i.d., 1.8 μm (Waters, USA). LC − MS/MS spectra were acquired using Waters SYNAPT G2-Si combined with ultra-performance liquid chromatography (Waters, USA).

### 2.3 Soybean germination

Germination of yellow soybean and black soybean was carried out according to the following method: prior to germination, both soybeans were carefully selected to eliminate any damaged and stained seeds or foreign materials. Seeds were rinsed with water before they were soaked in water at room temperature (25 °C), and then they were placed in a breathable container covered with wet gauze. The container was placed in the dark to germinate at room temperature. The seeds were rinsed with water twice every day during germination. When the sprout length grew to 0.5 cm, 1.0 cm, 1.5 cm, 2.0 cm, 4.0 cm, 8.0 cm, 12.0 cm, and 16.0 cm, the samples were removed and dried at 60 °C. The information of the samples was shown in Table S1. The germination images of yellow soybean and black soybean obtained with different sprout lengths after sprouting are shown in Fig. S3. The yields of samples were calculated. Five replicates were conducted for each sample.

### 2.4 Sample preparation procedure

The dry sample was crushed and powder that passed 65-mesh screen was collected and weighed. Aliquots of 25 mL of 70% methanol solution were added into the powder and heated reflux extraction for 2 h. The extract was filtered by 0.22 μm microporous membrane.

### 2.5 Standard solutions and quality control (QC)

A standard stock solution of each compound with a concentration of 1.00 mg/mL was prepared using dimethyl sulfoxide (DMSO), respectively. A mixed standard stock solution was further prepared with methanol at different concentrations. The mixed standard stock solutions simultaneously containing nine soybean isoflavones were then diluted with methanol to prepare a series of mixed working standard solutions. All these solutions were stored at −20 °C prior to use.

### 2.6 UPLC−DAD conditions

The detection wave length was set at 254 nm. The column used was an ACQUITY UPLC®HSS 2.1 mm × 100 mm i.d., 1.8 μm with 0.1% formic acid in water (A) and acetonitrile (B) as mobile phase. Solvent A and B gradient was as follows: 0.0–1.0 min, A from 95% to 89%; 1.0–2.0 min, A from 89% to 87%; 2.0–11 min, A from 87% to 75%; 11–15 min, A 75%; 15–20 min, A from 75% to 74%; 20–26 min, A from 74% to 65%; 26–27 min, A from 65% to 5.0%; 27–32 min, A 5.0%. The flow rate was 0.4 mL/min, and the column temperature was controlled at 30 °C. The injection volume was set to be 5 μL.

### 2.7 LC–QTOF–MS/MS conditions

The conditions for LC–QTOF–MS/MS analysis used an ACQUITY UPLC®HSS 2.1 mm × 100 mm i.d., 1.8 μm with solvent A (0.1% formic acid in water, LC − MS grade) and B (acetonitrile, LC − MS grade) as mobile phase. The gradient was identical to those used for UPLC–DAD. For LC–QTOF–MS/MS, both positive and negative ion modes were selected to further characterize nine soybean isoflavones with the full scan mass spectra from *m/z* 50 to 1200 Da. The capillary temperature and capillary voltage were maintained at 120 °C and 2 kV, respectively.

### 2.8 Method validation for UPLC–DAD method

According to those corresponding guidelines in Chinese Pharmacopoeia (2020 edition) and the established procedures in our group (Fu, Yu, Wang, & Qiu, 2020; Qiu, Shi, Wang, Wu, & Wang, 2018), the currently established UPLC–DAD method was validated in terms of selectivity, linear range, limit of detection (LOD), limit of quantification (LOQ), precision, accuracy, repeatability, stability, and recovery.

#### 2.8.1 Selectivity

The selectivity of the method was evaluated by comparing the chromatograms of blank solvent, mixed standard solution and typical samples.

#### 2.8.2 Linear range

The calibration curves were plotted using the concentration (X) of each analyte as abscissa and the peak area (Y) as ordinate. According to the calibration curve, the linear range of each compound was obtained.

#### 2.8.3 LOD and LOQ

The limit of detection (LOD) and limit of quantification (LOQ) of each analyte were determined as the concentrations at signal-to-noise ratios of 3 and 10, respectively.

#### 2.8.4 Precision and accuracy

Precision and accuracy were evaluated by assessing QC samples at three different concentration levels.

#### 2.8.5 Repeatability

The repeatability of the method was evaluated by analyzing the solutions of six identical samples prepared in parallel, and the relative standard deviation (RSD, %) value represented repeatability.

#### 2.8.6 Stability

One sample solution was prepared and determined at 0, 4, 6, 8, 12, and 24 h, and the peak area of each soybean isoflavone was recorded. The stability of each soybean isoflavone was investigated by the variation of peak area.

#### 2.8.7 Recovery

The recovery assessed by adding three different concentrations standard solution (approximately 80%, 100% and 120% of the original content of each soybean isoflavone contained in sample) to the sample of known concentration. The results were calculated as follows: Recovery = (amount found − original amount)/spiked amount × 100%.

### 2.9 Statistical analysis

In this present study, one-way analysis of variance was performed to explore the transformation profiles of the isoflavones in germinated soybean by using the statistical software package IBM SPSS 26.0 (IBM, Chicago, IL, USA).

## 3 Results and discussion

### 3.1 Chromatographic optimization

**Fig. 1:**
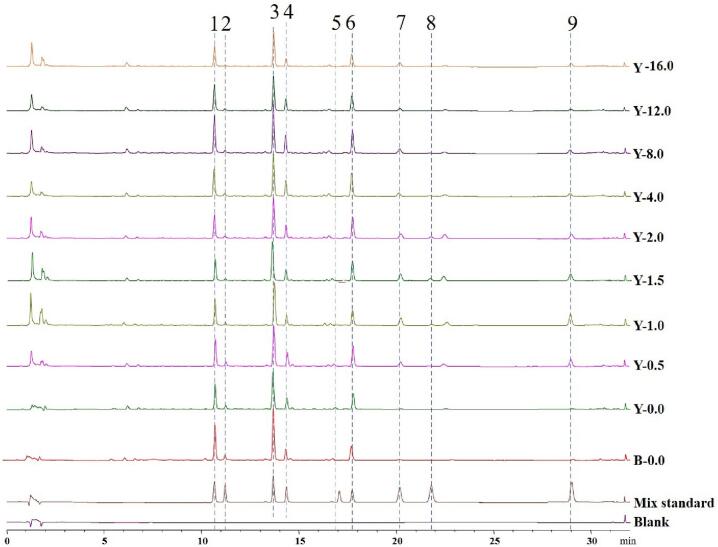
Typical UPLC−DAD chromatograms of nine soybean isoflavones in blank solvent, mixed standard solution and germinated soybean samples with different sprout length. 1: daidzin; 2: glycitin; 3: genistin; 4: 6”-*O*-malonyldaidzin; 5: 6”-*O*-acetylglycitin; 6: 6”-*O*-malonylgenistin; 7: daidzein; 8: glycitein; 9: genistein.

There are four types of target compounds in this study, including malonyl isoflavone glycoside (6”-*O*-malonyldaidzin, 6”-*O*-malonylgenistin), acetyl isoflavone glycoside (6”-*O*-acetylglycitin), glucose isoflavone glycoside (daidzin, genistin, glycitin) and isoflavone aglycone (daidzein, genistein, glycitein). According to the structure analysis, four pairs of compounds may have similar chemical polarities, including daidzin and glycitin, genistin and 6”-*O*-malonyldaidzin, 6”-*O*-acetylglycitin and 6”-*O*-malonylgenistin, daidzein and glycitein. These pairs of compounds are predicted to be eluted very closely. Therefore, precise separation is necessary to accurately quantification. All the nine isoflavones analyzed in the present study own same skeleton structures, and their maximum absorption peaks are all about at 254 nm. Therefore, the detection wavelength was set at 254 nm as the only wavelength. An UPLC column (ACQUITY UPLC®HSS column, 2.1 mm × 100 mm i.d., 1.8 μm) was used to obtain good separation and sharp peak shapes. The gradient elution was also adopted because of the large polarity difference and the long separation of retention time of compounds. In addition, 0.1% formic acid was added to the aqueous phase to improve the wake of chromatographic peaks. And acetonitrile was selected as the organic phase, because the separation efficiency of acetonitrile was better than that of methanol. Fig. 1 shows the typical UPLC−DAD chromatograms of nine soybean isoflavones under this experimental method. All the nine compounds have been well separated with good peak shapes in a relatively short running time of 32 min.

### 3.2 Optimization of the extraction method

For the optimization of the extraction process, heating reflux and ultrasonic extraction were compared. It is indicated that the total content of soybean isoflavones in the samples extracted by heating reflux is higher than that by ultrasonic extraction. Moreover, the extraction method recorded in Chinese Pharmacopoeia (2020 edition) is heating reflux for the determination of soybean. Moreover, five different concentrations of methanol solution (50%, 60%, 70%, 80%, and 90%) were used into the same sample to optimize extraction solvent. It was demonstrated that 70% methanol was the best extraction efficiency among all of them. Optimized extraction condition for the bioactive components of germinated soybean samples was finalized to be heating reflux extraction for 2 h with 70% methanol.

### 3.3 Method validation

#### 3.3.1 Selectivity

Fig. 1 shows the typical UPLC−DAD chromatograms of nine soybean isoflavones in blank solvent, mixed standard solution and typical germinated soybean samples with different sprout length under this experimental method. Daidzin, glycitin, genistin, 6”-*O*-malonyldaidzin, 6”-*O*-acetylglycitin, 6”-*O*-malonylgenistin, daidzein, glycitein, genistein were eluted at 10.8 ± 0.1, 11.3 ± 0.1, 13.8 ± 0.1, 14.4 ± 0.1, 17.1 ± 0.1, 17.6 ± 0.1, 20.2 ± 0.1, 21.9 ± 0.1, and 29.1 ± 0.1 min, respectively. There were no apparent interferences with the peaks of nine analytes of interest, suggesting a good selectivity of the established method.

#### 3.3.2 Linear range, LOD and LOQ

The regression equation, linear range, LOD and LOQ of nine isoflavones were shown in Table 1. The correlation coefficients of nine soybean isoflavones were all >0.9995, indicating the good linear correlation. The LOD and LOQ values of nine soybean isoflavones varied from 0.004 to 0.030 μg/mL and 0.008 to 0.060 μg/mL, respectively, exhibiting they had good sensitivity.

**Table 1:** The regression equation, linear range, LOD and LOQ of nine soybean isoflavones determined by UPLC−DAD.

Compound	Regression equation	R	Linear range (μg/mL)	LOD (μg/mL)	LOQ (μg/mL)
Daidzin	Y = 82.05× + 72.96	1.0000	1.22–122	0.024	0.048
Glycitin	Y = 75.28× + 26.00	0.9999	1.10–110	0.027	0.055
Genistin	Y = 102.2× + 93.28	0.9999	1.20–120	0.006	0.012
6”-*O*-Malonyldaidzin	Y = 65.15×-29.88	1.0000	1.08–108	0.005	0.010
6”-*O*-Acetylglycitin	Y = 76.20×-0.026	0.9999	0.008–0.415	0.004	0.008
6”-*O*-Malonylgenistin	Y = 74.54×-29.29	1.0000	1.00–100	0.005	0.001
Daidzein	Y = 125.3× + 1.095	0.9998	0.515–20.6	0.005	0.010
Glycitein	Y = 120.6× + 0.842	0.9998	0.061–6.10	0.006	0.012
Genistin	Y = 156.2× + 1.804	0.9998	0.530–21.2	0.030	0.060

#### 3.3.3 Precision and accuracy

The RSDs of the precision ranged from 0.35% to 9.93% for intra-day analyses and 0.30% to 9.93% for inter-day analyses, while the accuracy for all the nine soybean isoflavones were ranged from 97.6% to 102.0%, and the results were shown in Table S2, indicating that it was accurate and precise.

#### 3.3.4 Repeatability and stability

The RSDs of repeatability were <5.85%, and the results of stability testing at different times showed that nine compounds had good stability under current conditions (Table S2).

#### 3.3.5 Recovery

The recoveries of nine compounds determined by the method were summarized inTable S3. The recoveries of the nine compounds were within the range of 97.3% - 110.0%, and the RSDs were acceptable, indicating that it was accurate for the quantification of compounds.

### 3.4 Yields of germinated soybean samples

As shown in Table 2 the yields of germinated samples were calculated. and decreased gradually with the increase of sprout length. When the sprout length was 1.0 cm, the yield of germinated yellow soybeans was 83.8 ± 2.92%, while the yield of germinated black soybeans was 85.6 ± 3.58%. When the sprout length reached to 16.0 cm, the yields of germinated yellow and black soybeans were 65.7 ± 12.4% and 79.7 ± 4.98%, respectively.

**Table 2:** The yields of germinated yellow and black soybeans (mean ± SD, *n* = 5).

Sample ID	Yield (%)	Sample ID	Yield (%)
Y − 0.0	100	B − 0.0	100
Y − 0.5	85.5 ± 2.46⁎⁎	B − 0.5	86.4 ± 4.12⁎⁎
Y − 1.0	83.8 ± 2.92⁎⁎	B − 1.0	85.6 ± 3.58⁎⁎
Y − 1.5	82.7 ± 2.27⁎⁎	B − 1.5	85.0 ± 3.53⁎⁎
Y − 2.0	82.9 ± 1.63⁎⁎	B − 2.0	84.6 ± 3.45⁎⁎
Y − 4.0	80.8 ± 3.72⁎⁎	B − 4.0	83.3 ± 3.42⁎⁎
Y − 8.0	78.1 ± 3.86⁎⁎	B − 8.0	81.6 ± 4.51⁎⁎
Y − 12.0	73.0 ± 5.52⁎⁎	B − 12.0	81.8 ± 4.98⁎⁎
Y − 16.0	65.7 ± 12.4⁎⁎	B − 16.0	79.7 ± 4.98⁎⁎

⁎⁎ : compared with the weight before germination, *P* < 0.01. Y: yellow soybean; B: black soybean.

### 3.5 Identification of nine isoflavones in germinated soybean

In this study, a generic LC–QTOF–MS/MS method was used for the qualitative analysis of nine isoflavones without validation. According to the required retention time, accurate mass, MS/MS spectrometry fragment ions, and comparing these data with reference standards, nine soybean isoflavones were confirmed. The MS data of nine soybean isoflavones in germinated soybean by LC–QTOF–MS/MS were shown in Table 3. The information on the fragmentation of nine soybean isoflavones were essentially in agreement with the data reported in the literatures (Chan, Lin, Yu, Liu, & Fuh, 2006; Chen, Wu, Zhang, & Lin, 2012; Stürtz, Lander, Schmid, & Winterhalter, 2006**;** Wu, Wang, & Simon, 2003**)**. Its predominant molecular ions was [M + H]^+^, and the fragment ions of isoflavone glucoside, isoflavone malonyl glycoside and isoflavone acetyl glycoside were [M + H–glucose]^+^, [M + H–malonyl glucoside]^+^ and [M + H–acetyl glucoside]^+^.

**Table 3:** MS data of nine soybean isoflavones confirmed by LC–QTOF–MS/MS.

Compound	t_R_ (min)	Formula	Theoretical mass *(m/z)*	Measured mass *(m/z)*	Error (ppm)	Product ion mass (*m/z*)
Daidzin	5.04	C_21_H_20_O_9_	417.1180	417.1190	2.40	255.0656 [M + H–glucose]^+^
Glycitin	5.48	C_22_H_22_O_10_	447.1286	447.1275	−2.46	285.0764 [M + H–glucose]^+^
Genistin	7.30	C_21_H_20_O_10_	433.1129	433.1115	−3.23	271.0613 [M + H–glucose]^+^
6”-*O*-Malonyldaidzin	8.01	C_24_H_22_O_12_	503.1184	503.1190	1.19	255.0656 [M + H–malonyl glucoside]^+^
6”-*O*-Acetylglycitin	10.02	C_24_H_24_O_11_	489.1391	489.1397	1.23	285.0764 [M + H–acetyl glucoside]^+^
6”-*O*-Malonylgenistin	10.29	C_24_H_22_O_13_	519.1133	519.1134	0.19	271.0613 [M + H–malonyl glucoside]^+^
Daidzein	11.25	C_15_H_10_O_4_	255.0652	255.0656	1.57	−
Glycitein	12.03	C_16_H_12_O_5_	285.0758	285.0764	2.32	−
Genistin	16.28	C_15_H_10_O_5_	271.0601	271.0613	4.42	−

### 3.6 Transformation profiles of the isoflavones in germinated soybean

The contents of daidzin, glycitin, genistin, 6”-*O*-malonyldaidzin, 6”-*O*-acetylglycitin, 6”-*O*-malonylgenistin, daidzein, glycitein, genistein during germination were determined by UPLC–DAD method shown in Table 4, and the results were corrected using the yields provided in Table 2.

**Table 4:** The contents of nine soybean isoflavones in germinated soybean samples with different sprout length determined by UPLC−DAD (n = 5).

Sample ID	Content (nmol/g)								
	Daidzin	Glycitin	Genistin	6”-*O*-Malonyldaidzin	6”-*O*-Acetylglycitin	6”-*O*-Malonylgenistin	Daidzein	Glycitein	Genistein
Y–0.0	2029 ± 16.6	297 ± 10.4	2721 ± 106	877 ± 33.7	14.4 ± 0.46	1486 ± 127	140 ± 20.4	15.5 ± 0.56	143 ± 3.04
Y–0.5	1574 ± 154	246 ± 9.13	1871 ± 193	930 ± 123	3.59 ± 0.35	1440 ± 199	324 ± 151	38.8 ± 19.0	392 ± 158
Y–1.0	1306 ± 150	159 ± 20.8	1667 ± 145	701 ± 121	2.58 ± 1.75	1182 ± 189	710 ± 182	101 ± 22.8	701 ± 185
Y–1.5	1274 ± 194	152 ± 75.8	1618 ± 132	727 ± 151	2.13 ± 3.56	1217 ± 168	677 ± 175	94.5 ± 66.9	663 ± 98.5
Y–2.0	1329 ± 181	148 ± 4.99	1694 ± 327	756 ± 159	4.08 ± 4.67	1270 ± 334	564 ± 80.5	78.7 ± 9.15	490 ± 176
Y–4.0	1698 ± 153	163 ± 15.1	1982 ± 76.7	1038 ± 62.8	2.43 ± 1.43	1606 ± 41.6	302 ± 123	35.6 ± 19.2	191 ± 112
Y–8.0	1532 ± 494	100 ± 58.2	1808 ± 266	850 ± 214	1.97 ± 1.28	1322 ± 145	544 ± 188	49.2 ± 13.9	381 ± 124
Y–12.0	1365 ± 511	86.7 ± 59.7	1742 ± 287	684 ± 233	1.33 ± 1.09	1125 ± 217	547 ± 201	36.1 ± 13.9	392 ± 235
Y–16.0	1278 ± 308	71.9 ± 31.5	1749 ± 245	574 ± 72.9	1.44 ± 1.25	985 ± 50.6	599 ± 142	40.8 ± 10.8	385 ± 160
B–0.0	2256 ± 99.0	434 ± 29.1	3116 ± 127	831 ± 31.4	18.5 ± 1.04	1317 ± 139	127 ± 15.3	8.70 ± 0.75	186 ± 5.23
B–0.5	1370 ± 103	360 ± 37.0	1606 ± 144	832 ± 89.5	8.76 ± 4.09	1217 ± 145	154 ± 22.0	18.4 ± 6.27	223 ± 33.4
B–1.0	1189 ± 96.7	233 ± 33.4	1444 ± 112	702 ± 47.4	6.02 ± 2.91	1091 ± 86.0	401 ± 72.6	108 ± 25.1	408 ± 64.6
B–1.5	1197 ± 107	212 ± 50.8	1441 ± 115	699 ± 119	5.99 ± 3.14	1065 ± 173	435 ± 123	117 ± 46.9	399 ± 81.1
B–2.0	1267 ± 180	216 ± 59.0	1487 ± 140	790 ± 167	6.27 ± 2.76	1161 ± 184	337 ± 165	91.3 ± 56.7	284 ± 114
B–4.0	1468 ± 106	254 ± 24.5	1551 ± 76.2	993 ± 32.0	5.65 ± 3.62	1330 ± 45.1	160 ± 42.6	33.8 ± 11.8	122 ± 25.4
B–8.0	1441 ± 401	216 ± 90.6	1481 ± 211	905 ± 220	7.22 ± 4.88	1186 ± 185	261 ± 202	49.4 ± 45.8	189 ± 188
B–12.0	1329 ± 364	143 ± 82.9	1518 ± 308	762 ± 238	4.37 ± 3.19	1117 ± 305	425 ± 155	63.4 ± 23.8	287 ± 161
B–16.0	1196 ± 255	129 ± 69.1	1374 ± 237	694 ± 215	3.37 ± 2.00	1022 ± 284	458 ± 238	63.1 ± 25.6	312 ± 192

**Fig. 2:**
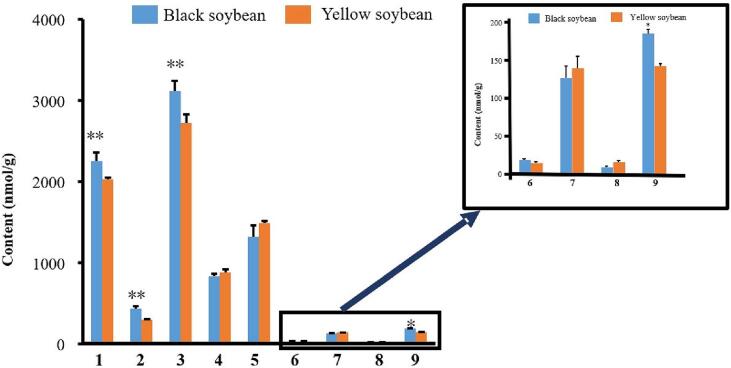
Comparison of the contents of nine soybean isoflavones in yellow and black soybean samples. **: compared with yellow soybean, P < 0.01; *: compared with yellow soybean, *P* < 0.05. 1: daidzin; 2: glycitin; 3: genistin; 4: 6”-*O*-malonyldaidzin; 5: 6”-*O*-malonylgenistin; 6: 6”-*O*-acetylglycitin; 7: daidzein; 8: glycitein; 9: genistein. (For interpretation of the references to colour in this figure legend, the reader is referred to the web version of this article.)

**Fig. 3:**
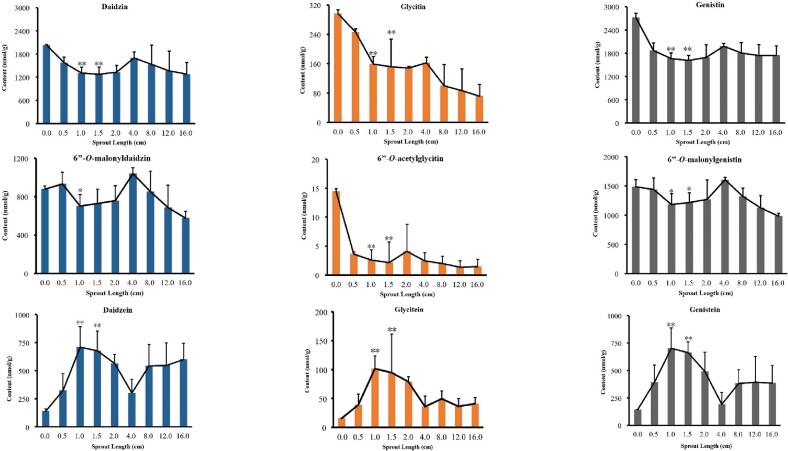
The determined contents of nine isoflavones in germinated yellow soybean (*n* = 5). **: compared with yellow soybean, *P* < 0.01; *: compared with yellow soybean, *P* < 0.05. (For interpretation of the references to colour in this figure legend, the reader is referred to the web version of this article.)

**Fig. 4:**
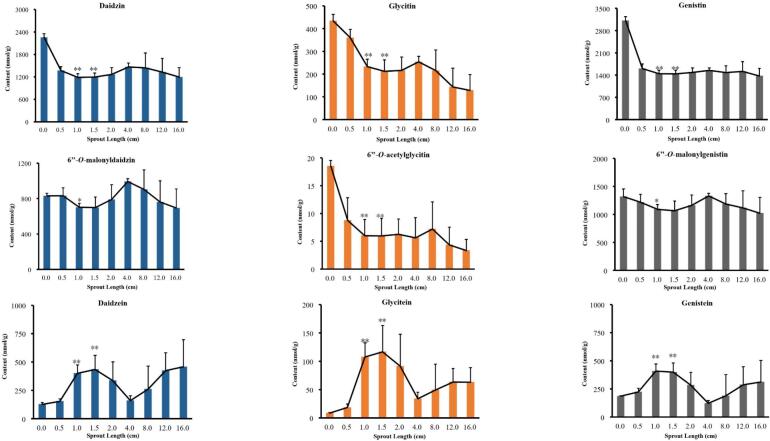
The determined contents of nine isoflavones in germinated black soybean (n = 5). **: compared with yellow soybean, *P* < 0.01; *: compared with yellow soybean, *P* < 0.05. (For interpretation of the references to colour in this figure legend, the reader is referred to the web version of this article.)

Fig. 2 showed the comparison of the contents of nine soybean isoflavones in yellow and black soybean samples. Fig. 3 and Fig. 4 provided the determined contents of nine isoflavones in germinated soybeans, respectively.

The results in Fig. 2 showed that the contents of daidzein, glycitein, genistein and genistin in black soybeans were significantly higher than those in yellow soybeans before germination (*P* < 0.05). However, after the soybean is germinated, the contents of daidzein and genistein in the yellow soybeans are all higher than those in the black soybeans (*P* < 0.05). The contents of daidzein and genistein in the germinated yellow soybeans are about 160% of those in the germinated black soybeans. It is suggested that yellow soybeans should be selected as the raw material for germination and processing.

Among the isoflavones examined in the work, the content of daidzein, glycitein, genistein showed a significant increase at the sprout length from 0.0 to 1.5 cm (*P* < 0.05), and then decreased rapidly when the sprout length at 1.5–4.0 cm (*P* < 0.05). The contents of them reached the peak at sprout length 1.0–1.5 cm. When the sprout length was in the range of 4.0–16.0 cm, the content of these compounds tended to stable. As shown in Fig. 3 and Fig. 4, the contents of daidzin, glycitin, genistein and 6”-*O*-acetylglycitin were the highest when they were not germinated, and decreased slightly with the growth of sprout length. And The contents of 6”-*O*-malonyldaidzin, and 6”-*O*-malonylgenistin were the highest when the sprout length was 4.0 cm, but it was not much different from that when the sprout length was 1.0–1.5 cm.

In another study (Hu et al., 2020) soybeans were germinated at 25 °C for 168 h, and the results showed that the contents of daidzin, genistin and glycitein decreased, while the contents of other components, genistein, daidzein, 6”-*O*-malonyldaidzin, and 6”-*O*-malonylgenistin increased. However, their research didn't discuss it from the angle of sprout length, ignoring the content changes of some active ingredients. For example, in our research, when the sprout length was 1.0–1.5 cm, it can not only shorten the germination time of soybean, but also get better efficacy. Although the contents of other components have increased compared with those without germination, they are almost the same as those when the sprout length is 1.0–1.5 cm. Therefore, to save time and cost, soybean with the sprout length of 1.0–1.5 cm could be selected.

## 4 Conclusion

A selective and accurate UPLC–DAD method has been established and validated, which has been successfully applied for the quantification of daidzin, glycitin, genistin, 6”-*O*-malonyldaidzin, 6”-*O*-acetylglycitin, 6”-*O*-malonylgenistin, daidzein, glycitein and genistein. An LC–QTOF–MS/MS method was also applied for the identification of these compounds to ensure the quantification accurate.

As we all known, the selection of raw materials also needs to consider the contents of active components after germination, so yellow soybeans may be more suitable. When soybean was germinated at the sprout length 1.0–1.5 cm, the contents of daidzein, glycitein and genistein were all at high levels. For those who need an estrogen supplement, it may be better to take germinated soybeans with the sprout length at 1.0–1.5 cm. In conclusion, the differences in the contents of the active compounds in germinated soybeans highlight to some extent the importance of germinated soybean with the sprout length 1.0–1.5 cm as a promising functional food source.

## CRediT authorship contribution statement

**Hong Chen:** Project administration, Methodology, Formal analysis, Data curation. **Rezeye Aili:** Writing – original draft, Formal analysis, Data curation. **Manyuan Wang:** Writing – review & editing, Investigation, Conceptualization. **Feng Qiu:** Writing – review & editing, Supervision, Resources, Funding acquisition.

## Declaration of competing interest

The authors declare that they have no known competing financial interests or personal relationships that could have appeared to influence the work reported in this paper.

## Data Availability

Data will be made available on request.

## Appendix A Supplementary data

Supplementary material Image 1
