# Supplementary material for: Transformation profiles of the isoflavones in germinated soybean based on UPLC–DAD quantification and LC–QTOF–MS/MS confirmation
Source: Food Chem X. 2024 Apr 24;22:101413. doi: 10.1016/j.fochx.2024.101413 (PMC11068514; doi:10.1016/j.fochx.2024.101413)
Supplement: Supplementary file 1 — Supplementary material [file mmc1.docx]

**Supplementary material captions**

**Fig. S1** The proposed biosynthesis pathway of main isoflavones contained in soybean

**Fig. S2** Chemical structures of main isoflavones contained in soybean

**Fig. S3** The germination images of yellow soybean and black soybean obtained with different sprout lengths after sprouting

**Table S1** The information of different germinated soybean samples

**Table S2** Precision, accuracy, repeatability and stability results of nine soybean isoflavones determined by UPLC−DAD (n=6)

**Table S3** Recovery of nine soybean isoflavones determined by UPLC−DAD (n=6)

**Fig. S1** The proposed biosynthesis pathway of main isoflavones contained in soybean

**
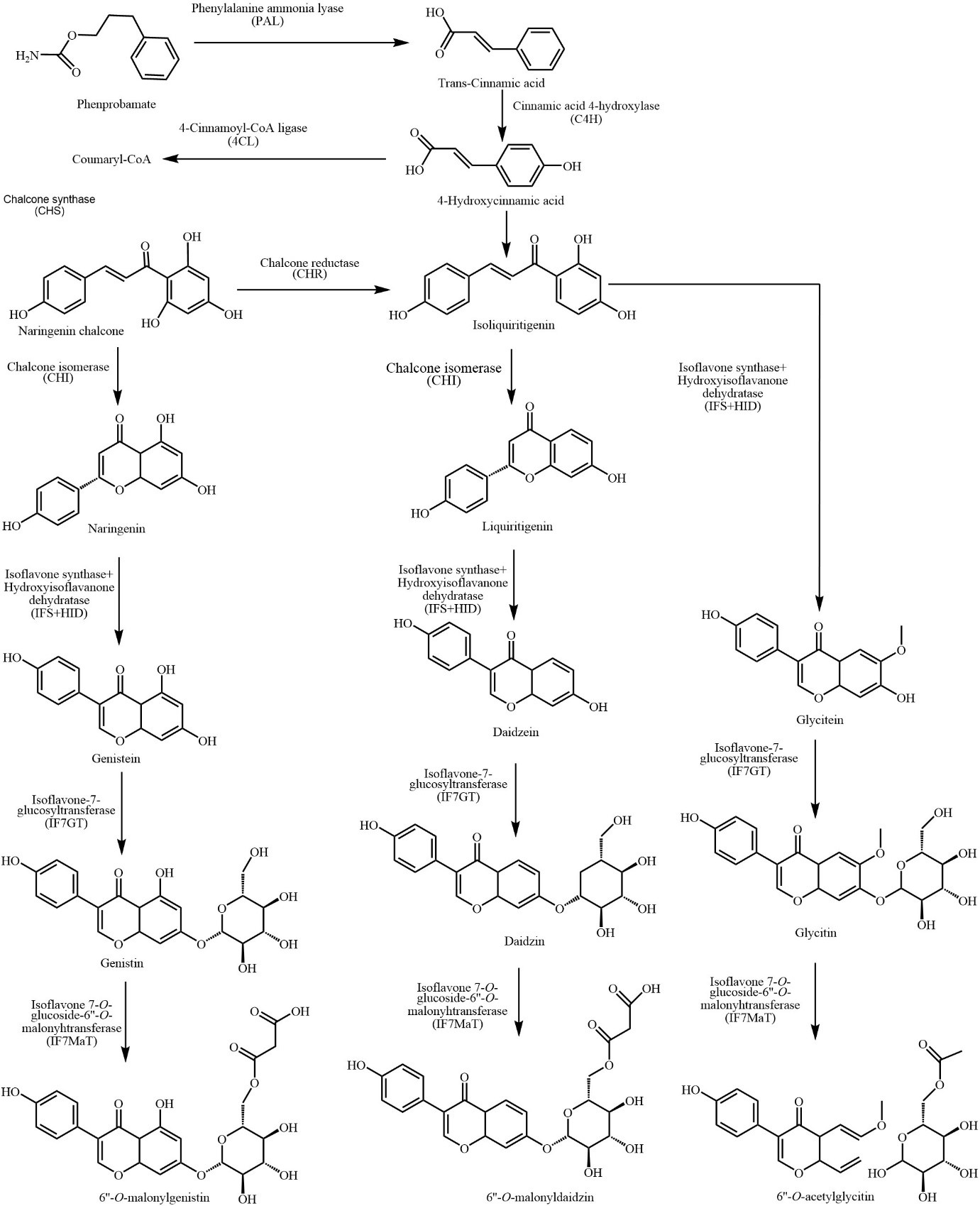
**

**Fig. S2** Chemical structures of main isoflavones contained in soybean

**
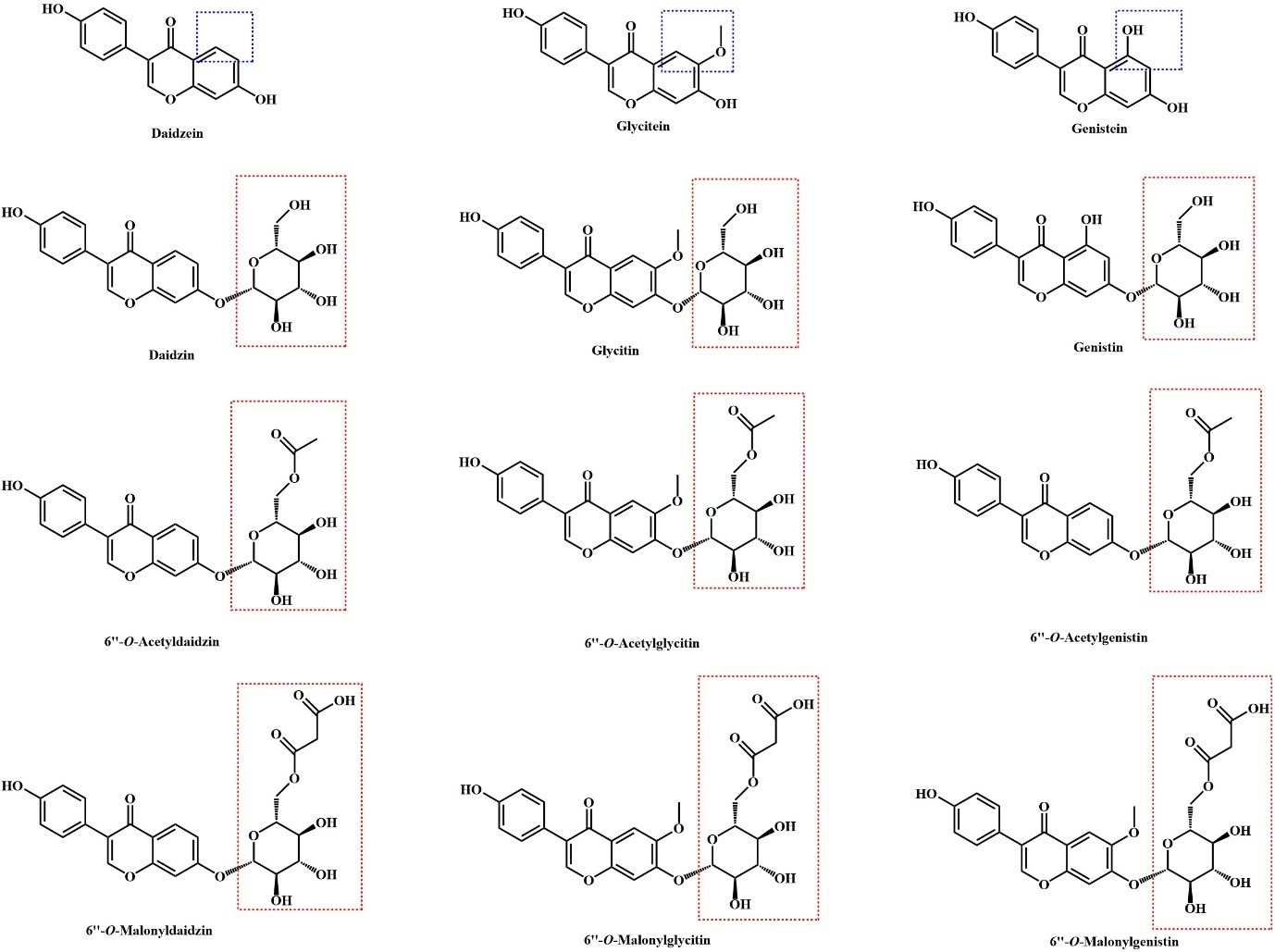
**

**Fig. S3** The germination images of yellow soybean and black soybean obtained with different sprout lengths after sprouting

**
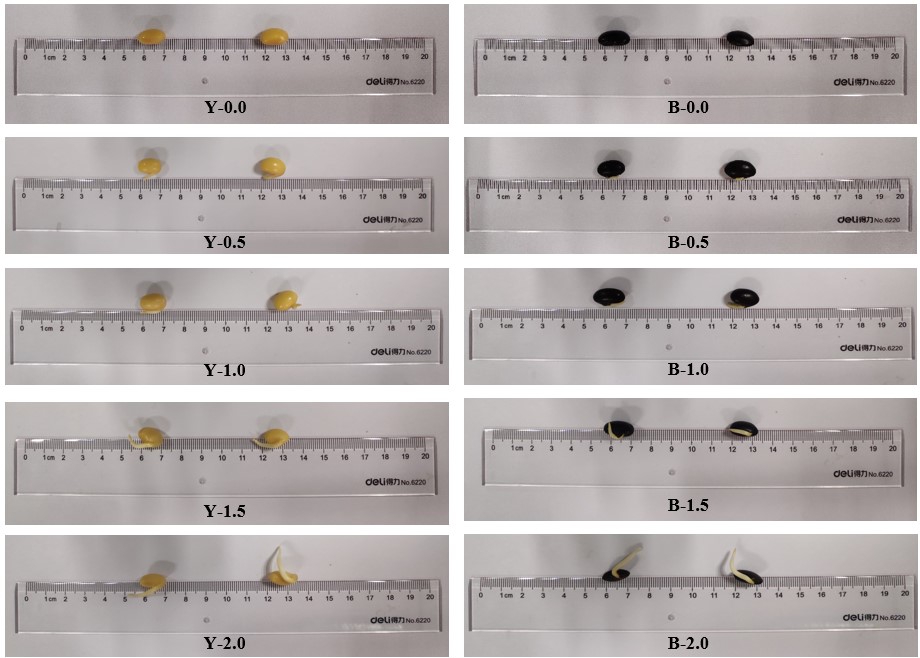
**

**
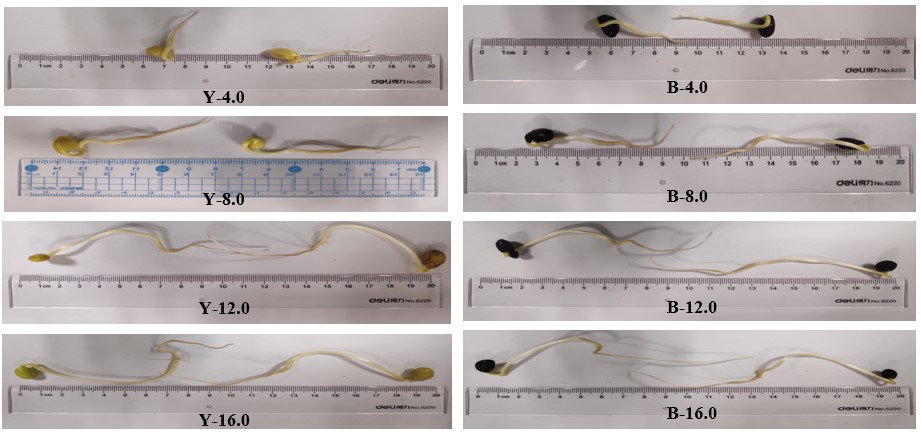
**

**Table S1** The information of different germinated soybean samples

| **Sample ID** | **Description** |
| --- | --- |
| Y-0.0 | Yellow soybean (raw material) |
| Y-0.5 | Soybean with the sprout length of 0.5 cm |
| Y-1.0 | Soybean with the sprout length of 1.0 cm |
| Y-1.5 | Soybean with the sprout length of 1.5 cm |
| Y-2.0 | Soybean with the sprout length of 2.0 cm |
| Y-4.0 | Soybean with the sprout length of 4.0 cm |
| Y-8.0 | Soybean with the sprout length of 8.0 cm |
| Y-12.0 | Soybean with the sprout length of 12.0 cm |
| Y-16.0 | Soybean with the sprout length of 16.0 cm |
| B-0.0 | Black soybean (raw material) |
| B-0.5 | Black soybean with the sprout length of 0.5 cm |
| B-1.0 | Black soybean with the sprout length of 1.0 cm |
| B-1.5 | Black soybean with the sprout length of 1.5 cm |
| B-2.0 | Black soybean with the sprout length of 2.0 cm |
| B-4.0 | Black soybean with the sprout length of 4.0 cm |
| B-8.0 | Black soybean with the sprout length of 8.0 cm |
| B-12.0 | Black soybean with the sprout length of 12.0 cm |
| B-16.0 | Black soybean with the sprout length of 16.0 cm |

**Table S2** Precision, accuracy, repeatability and stability results of nine soybean isoflavones determined by UPLC−DAD (n=6)

| **Compound** | **Concentration (μg/mL)** | **Precision (n=6)** | | **Accuracy (%)** | **Repeatability (RSD, %)** | **Stability (RSD, %)** |
| --- | --- | --- | --- | --- | --- | --- |
|  |  | **Intra-day (RSD, %)** | **Inter-day (RSD, %)** |  |  |  |
| Daidzin | 2.44 | 2.20 | 1.65 | 99.0 | 2.87 | 1.39 |
|  | 12.2 | 3.97 | 3.54 | 98.1 |  |  |
|  | 97.6 | 2.51 | 1.71 | 97.6 |  |  |
| Glycitin | 2.20 | 0.79 | 0.74 | 101 | 1.83 | 0.00 |
|  | 11.0 | 2.78 | 2.56 | 102 |  |  |
|  | 88.0 | 2.69 | 1.71 | 101 |  |  |
| Genistin | 2.40 | 2.39 | 2.24 | 97.9 | 2.84 | 2.37 |
|  | 12.0 | 4.96 | 4.53 | 101 |  |  |
|  | 96.0 | 0.81 | 0.60 | 102 |  |  |
| 6’’-*O*-Malonyldaidzin | 2.16 | 1.31 | 2.32 | 101 | 2.80 | 0.42 |
|  | 10.8 | 6.26 | 5.30 | 99.9 |  |  |
|  | 86.4 | 0.75 | 1.56 | 101 |  |  |
| 6’’-*O*-Acetylglycitin | 0.02 | 9.93 | 9.93 | 100 | 5.84 | 1.07 |
|  | 0.04 | 6.83 | 5.82 | 100 |  |  |
|  | 0.33 | 6.65 | 4.82 | 98.7 |  |  |
| 6’’-*O*-Malonylgenistin | 2.00 | 2.25 | 2.75 | 102 | 4.04 | 0.05 |
|  | 10.0 | 1.33 | 1.40 | 100 |  |  |
|  | 80.0 | 1.97 | 1.36 | 102 |  |  |
| Daidzein | 1.03 | 1.54 | 2.49 | 98.0 | 2.87 | 1.39 |
|  | 4.12 | 4.08 | 2.41 | 101 |  |  |
|  | 16.5 | 5.00 | 3.28 | 100 |  |  |
| Glycitein | 0.12 | 3.84 | 2.37 | 102 | 1.75 | 2.01 |
|  | 0.61 | 4.14 | 2.73 | 99.2 |  |  |
|  | 4.88 | 0.65 | 0.30 | 100 |  |  |
| Genistin | 1.06 | 1.77 | 1.55 | 102 | 4.04 | 3.00 |
|  | 4.24 | 4.64 | 2.94 | 101 |  |  |
|  | 17.0 | 0.35 | 0.39 | 102 |  |  |

**Table S3** Recovery of nine soybean isoflavones determined by UPLC−DAD (n=6)

| **Compound** | **Added amount (%)** | **Recovery (%)** | **RSD (%)** |
| --- | --- | --- | --- |
| Daidzin | 80 | 102 | 2.26 |
|  | 100 | 99.5 | 1.17 |
|  | 120 | 102 | 2.66 |
| Glycitin | 80 | 101 | 4.27 |
|  | 100 | 102 | 2.36 |
|  | 120 | 106 | 3.19 |
| Genistin | 80 | 108 | 1.02 |
|  | 100 | 107 | 1.49 |
|  | 120 | 107 | 1.68 |
| 6’’-*O*-Malonyldaidzin | 80 | 106 | 2.20 |
|  | 100 | 106 | 1.33 |
|  | 120 | 105 | 1.24 |
| 6’’-*O*-Acetylglycitin | 80 | 99.9 | 2.37 |
|  | 100 | 103 | 3.07 |
|  | 120 | 101 | 5.47 |
| 6’’-*O*-Malonylgenistin | 80 | 108 | 0.62 |
|  | 100 | 106 | 2.47 |
|  | 120 | 108 | 1.50 |
| Daidzein | 80 | 108 | 0.75 |
|  | 100 | 107 | 1.98 |
|  | 120 | 110 | 3.46 |
| Glycitein | 80 | 97.3 | 2.25 |
|  | 100 | 98.2 | 6.28 |
|  | 120 | 97.3 | 5.62 |
| Genistein | 80 | 103 | 2.29 |
|  | 100 | 102 | 4.82 |
|  | 120 | 104 | 3.62 |
